# Fine-grained interplanetary dust input during the Turonian (Late Cretaceous): evidence from osmium isotope and platinum group elements

**DOI:** 10.1038/s41598-023-49252-5

**Published:** 2023-12-12

**Authors:** Hironao Matsumoto, Akira Ishikawa, Rodolfo Coccioni, Fabrizio Frontalini, Katsuhiko Suzuki

**Affiliations:** 1 Japan Agency for Marine-Earth Science and Technology (JAMSTEC), 2–15, Natsushima, Yokosuka, Kanagawa 237-0061 Japan; 2 Department of Earth and Planetary Sciences, Tokyo Institute of Technology, Tokyo, 152-8550 Japan; 3 University of Urbino Carlo Bo, 61029 Urbino, Italy; 4 DiSPeA, University of Urbino Carlo Bo, Campus Scientifico Enrico Mattei, Località Crocicchia, 61029 Urbino, Italy

**Keywords:** Palaeoceanography, Palaeoclimate

## Abstract

The Turonian age (~ 90–94 Ma) was the hottest geological interval in the Cretaceous and also marked by the K3 event, a pronounced enrichment of ^3^He in pelagic sediments (i.e., massive input of extraterrestrial materials). Here, we present Os isotopic (^187^Os/^188^Os) and platinum group element (PGE) data from Turonian sedimentary records. After a sharp unradiogenic shift during the end-Cenomanian oceanic anoxic event 2, the ^187^Os/^188^Os ratios declined continuously throughout the Turonian, which could be ascribed to the formations of several large igneous provinces (LIPs). Because the interval with the most unradiogenic ^187^Os/^188^Os ratios (i.e., enhanced LIP volcanism) does not correspond to the warmest interval during the mid-Cretaceous, additional sources of CO_2_, such as subduction zone volcanism or the kimberlite formation, may explain the Cretaceous Thermal Maximum. As Os isotope ratios do not show any sharp unradiogenic shifts and PGE concentrations do not exhibit a pronounced enrichment, an influx of fine-grained cosmic dust to the Earth’s surface, possibly from the long-period comet showers, can be inferred at the time of the ^3^He enrichment during the mid-Turonian K3 event. Our findings highlight the different behaviors of ^3^He and PGE information in the sedimentary rocks during the input of fined-grained extraterrestrial materials.

The mid-Cretaceous was one of the hottest geological intervals in the Phanerozoic and also experienced severe environmental perturbations, including the worldwide deposition of organic-rich sediments (oceanic anoxic events: OAEs) and extinctions of marine planktons^1–3^. The late Cenomanian to early Santonian interval was characterized by warm climatic conditions^4–6^. The warmest climate, called the Cretaceous Thermal Maximum (CTM), occurred from the late Cenomanian to the early Turonian^4–7^. Even at high latitudes (~ 60°S), sea-surface and -bottom temperatures estimated from δ^18^O_foram_ data were ~ 25–30 °C and ~ 20 °C, respectively^4–6,8^. TEX_86_ data also support extremely high temperatures in the southern high latitudes during the Turonian^8,9^. No continental ice sheets were present, even at the polar regions^2^, and the West Antarctica was covered by rich vegetation^10^. Such exceptionally global warmth would have been sustained by high *p*CO_2_ in the atmosphere of up to several thousand ppm^11,12^.

The Cenomanian–Turonian interval also experienced massive volcanic episodes during which large igneous provinces (LIPs), including the Caribbean Plateau, the Madagascar Flood Basalt Province, and the High Arctic Large Igneous Province (HALIP), were formed^13–15^. The Caribbean Plateau was emplaced in the paleo-Eastern Pacific^14^ (Fig. 1). Although the eastern part of this oceanic plateau has been subducted underneath the proto-Caribbean Ocean^16^, its aerial extent is estimated to be ~ 1.54 × 10^6^ km^2^^17^. The Madagascar Flood Basalt Province, located around Madagascar, is composed of basalt flows and dykes and some rhyolite flows (Fig. 1)^13^. The radiometric ages of the Caribbean Plateau (~ 97–70 Ma^14^) and Madagascar Flood Basalt Province (92–66 Ma^18–20^) roughly correspond to those of OAE2 (93.9 Ma, at the end of the Cenomanian) and the CTM (~ 100–90 Ma). Also, main volcanic pulses of HALIP occurred around 97 to 100 Ma^15^. Volcanic emissions of CO_2_ during the formation of these basaltic plateaus may have therefore contributed to the extremely warm climatic conditions and contemporaneous environmental perturbations^11^. Large uncertainties in the radiometric ages have however hampered the determination of precise chronological correlations between these volcanic events and the CTM.

**Figure 1:**
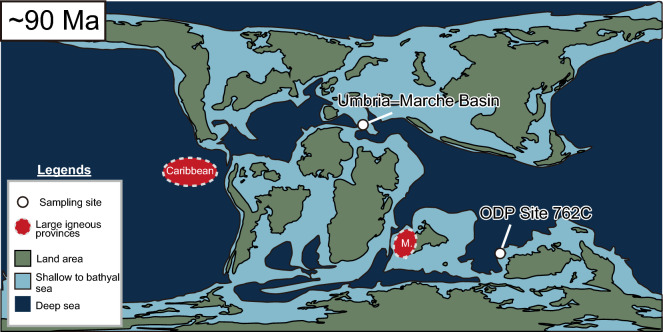
Paleo-geographical reconstruction at 91 Ma^69^. “M.” represents the position of the Madagascar Flood  Basalt Provinces. The map was created with Illustrator CS5.5 (https://www.adobe.com/products/illustrator.html).

One of the characteristic features of the Turonian age is the enhanced input of extraterrestrial materials. Farley et al.^21^ reported a pronounced enrichment of ^3^He in the sedimentary sequences deposited in the Tethys and Indian Oceans that they named the “K3 event” (Figs. 2 and 3). Because ^3^He is abundant in interplanetary dust particles (IDPs), they interpreted the cause of the K3 event to be a pronounced input of IDPs, possibly as lunar cosmic dust^21^. In a later study, Martin et al.^22^ reported an increase in rare types of extraterrestrial materials (i.e., vanadium-rich chrome spinel and ordinary H chondritic grains) in the Tethyan sedimentary record during the K3 event and proposed that the K3 event was caused by resonance ejection of small asteroids from different regions of the asteroid belt. Although several hypotheses have been proposed to explain the K3 event, a complete understanding of the event and its importance in Earth's environment and elemental cycles have been hampered by limited geochemical data^21,22^.

**Figure 2:**
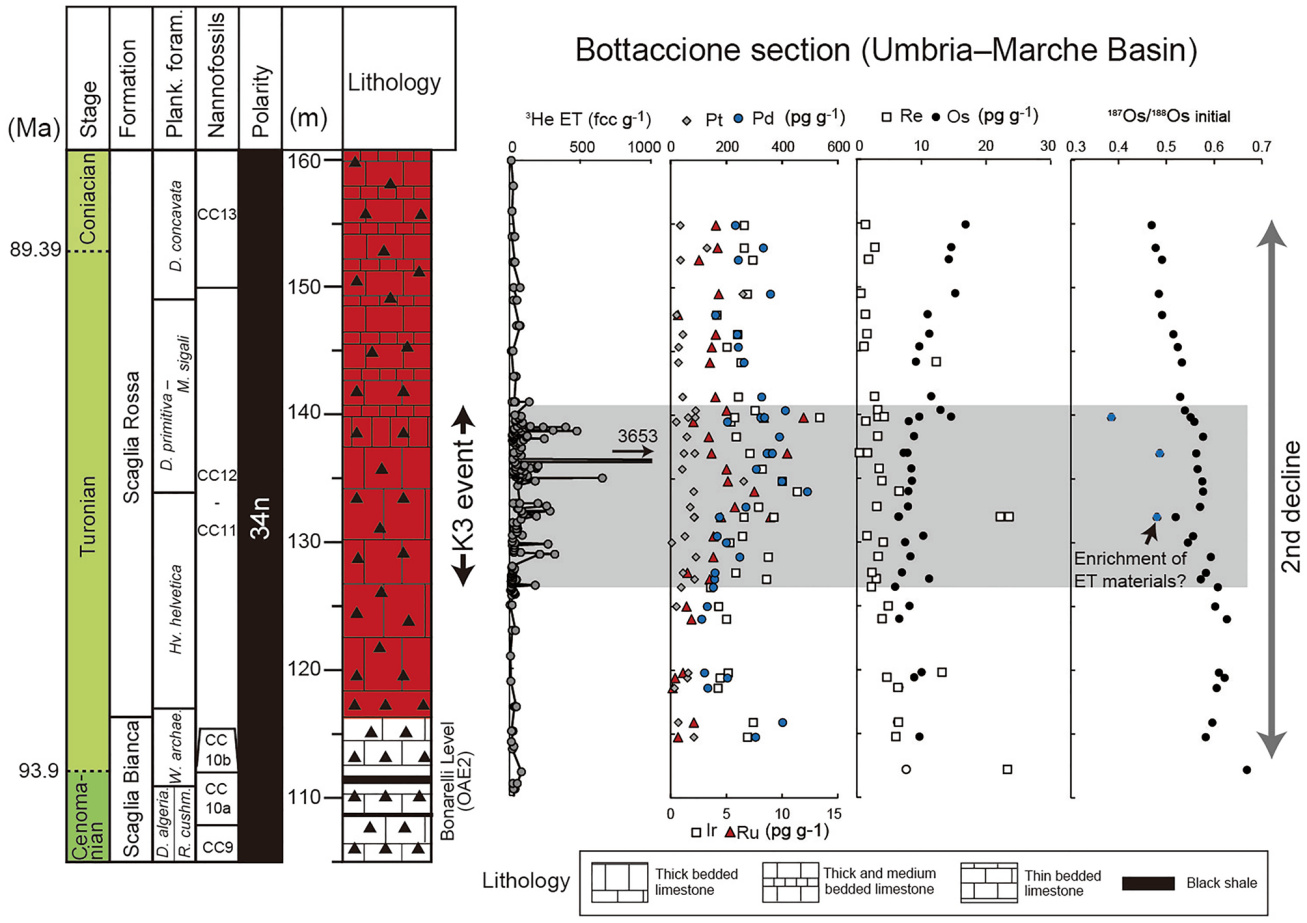
Geochemical record of the Bottaccione section (Umbria–Marche Basin, central Italy). The lithology and biostratigraphy are based on Ref.^26^. ^3^He concentration is based on Ref.^21^. Concentrations of platinum group elements and Os isotopic information are from this study. The stratigraphy reported in Ref.^21^ has been appropriately correlated to that of Ref.^26^. *algeria.*—*algeriana*, *archaeo.*—*archaeocretacea*, *cushm.*—*cushmani, D*.—*Dicarinella*, *Hv*.—*Helvetoglobotruncana*, *M*.—*Marginotruncana*, *R*.—*Rotalipora*, *W*.—*Whiteinella*, and ET—extraterrestrial.

**Figure 3:**
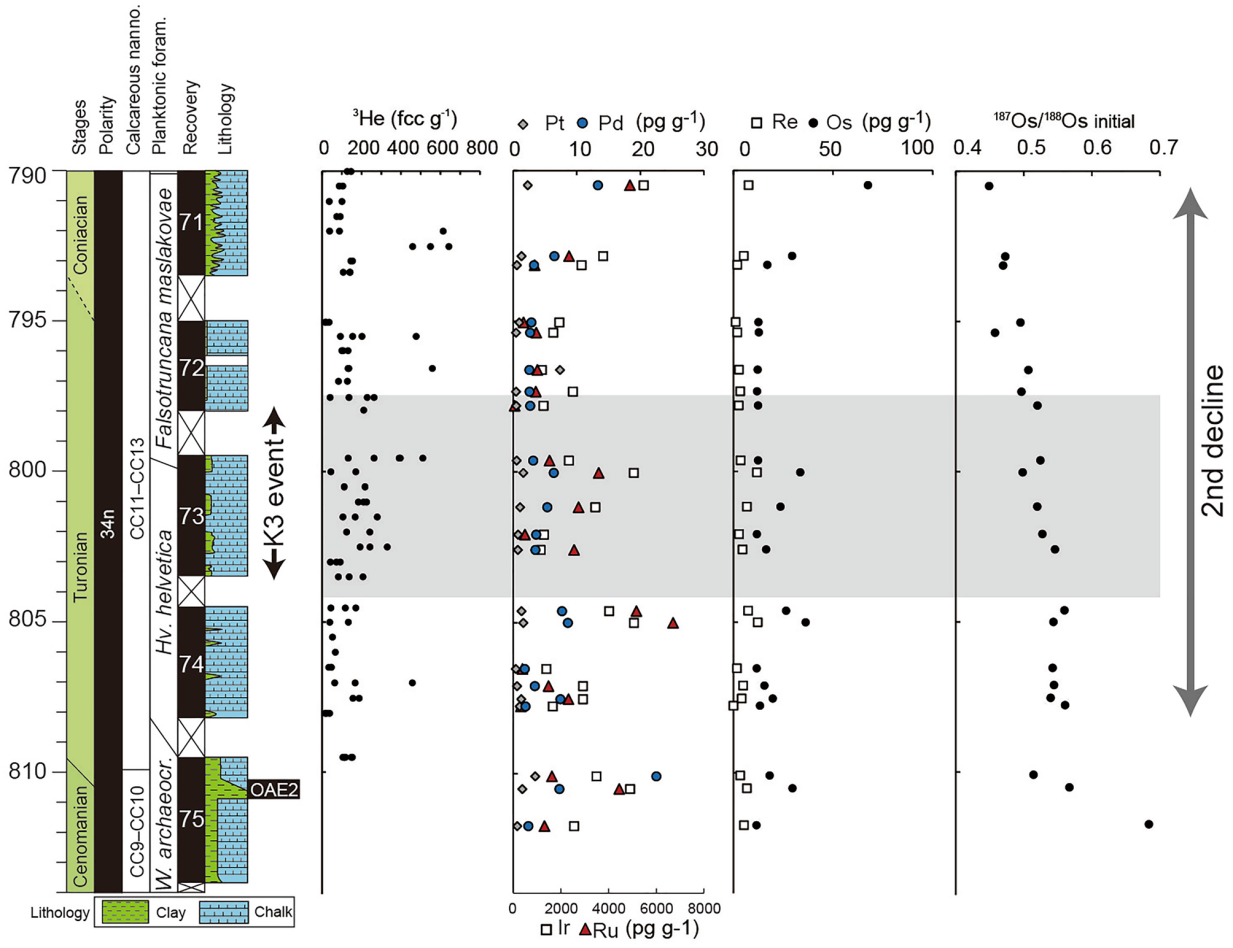
Geochemical record of the Ocean Drilling Program Site 762C (Exmouth Plateau), Indian Ocean. The lithology is from Ref.^27^. Biostratigraphy is from Ref.^27^. ^3^He information is from Ref.^21^. Concentrations of platinum group elements and Os isotopic information are from this study. *archaeocr*.—*archaeocretacea*, *Hv*.—*Helvetoglobotruncana*, and* W*.—*Whiteinella*.

Os isotopic (^187^Os/^188^Os) values of paleo-seawater recorded in sedimentary rock are the best proxy for estimating the influence of inputs of mantle-derived and extraterrestrial materials into the ocean. Os supplied from such materials has unradiogenic (low) values (~ 0.12), whereas Os supplied from continental sources has radiogenic (high) values (~ 1.5)^23^. The ^187^Os/^188^Os of seawater thus represents a balance between inputs from continental weathering and inputs of mantle-derived and extraterrestrial materials^23^. Therefore, ^187^Os/^188^Os variations can provide insight into the timing of enhanced inputs of mantle-derived materials (e.g., LIP volcanism) and extraterrestrial materials during the Turonian. In addition, patterns of platinum group elements (PGEs) (i.e., Os, Ir, Ru, Pt, and Pd) in sediments can also shed light on the flux of extraterrestrial materials, because PGEs are highly enriched in extraterrestrial materials and their chondrite-normalized pattern is distinctive from that of terrestrial materials^24,25^.

In this study, we reconstructed paleo-seawater Os isotopic variations and PGE patterns in pelagic sedimentary rocks collected from the Bottaccione section, deposited in the Tethyan Ocean, and from Ocean Drilling Program (ODP) Site 762C, which was deposited in the Indian Ocean (Fig. 1). We first inferred the cause of the CTM from the Os and Sr isotopic variations. Then, combining our new Os and PGE data with pre-existing ^3^He data, we re-examined the possible input of extraterrestrial materials during the K3 event.

## Geological settings

Sedimentary rocks samples were collected from the Bottaccione section in the Umbria–Marche Basin (central Italy) (Fig. 1). The sedimentary rocks of this basin were deposited in a pelagic setting in the central-western Tethys Ocean^26^. The sedimentary sequence of the studied interval consists of white to reddish limestone with repeated chert layers rich in planktonic foraminifera and calcareous nannofossils^25^. For Os and PGE analysis, we collected lower Turonian to lower Coniacian limestone samples.

Sedimentary rock samples were also collected from ODP Site 762C. These sediments were deposited on the central Exmouth Plateau in the Indian Ocean (Fig. 1). Most of the studied samples are composed of nannofossil chalk and clayey chalk containing planktonic foraminifera^27^. However, a distinctive brownish claystone layer at ~ 818 mbsf (Site 762C, Core 75, Sect. 2, ~ 130 cm) is considered to be the regional sedimentary expression of OAE 2 in the Indian Ocean^28^. For this study, we collected chalk and clayey chalk samples from the upper Cenomanian to lower Coniacian, but we did not collect samples from the OAE2 interval in this core because no material was available. We conducted Re-Os and PGE analyses of these sedimentary rock samples following Refs.^20,29,30^.

## Results

**Figure 4:**
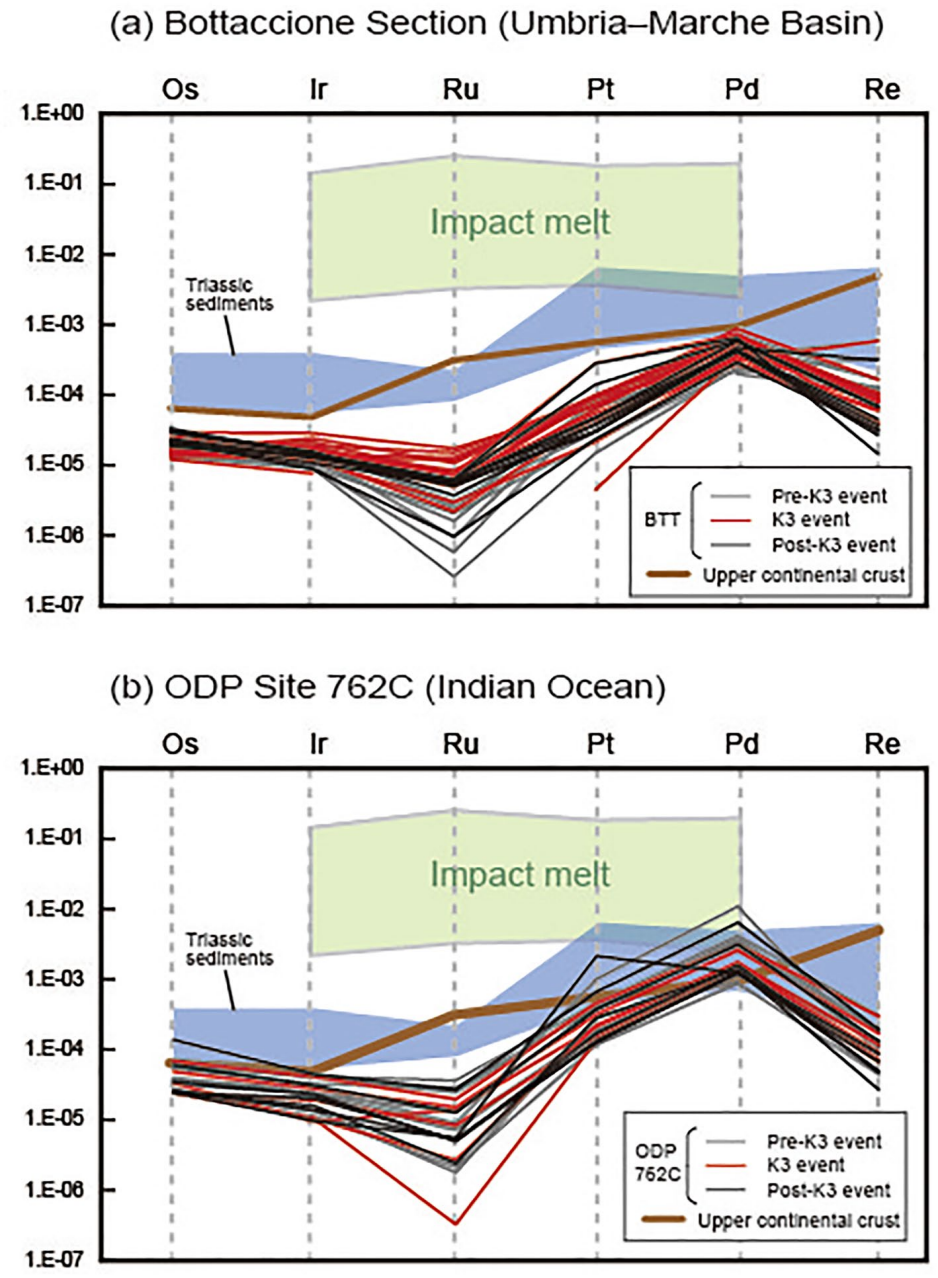
Chondrite-normalized PGE patterns at (**a**) Bottaccione section and (**b**) ODP Site 762C. The PGE concentrations of chondrite are from Ref.^70^. PGE concentrations of upper continental crust and Triassic sedimentary rock are from Refs.^25,30^, respectively. PGE concentrations of impact melt are from Ref.^71^.

The concentrations of Os, Ir, Ru, Pt, Pd, and Re in the Bottaccione section ranged from 6 to 16 pg g^−1^, 5 to 11 pg g^−1^, ~ 0 to 12 pg g^−1^, ~ 0 to 260 pg g^−1^, 111 to 491 pg g^−1^, and 0.5 to 23 pg g^−1^, respectively (Supplementary Table S1 and Fig. 2). The Os, Ir, Ru, Pt, Pd, and Re concentrations in rock samples from the core drilled at the ODP Site 762C varied from 11 to 64 pg g^−1^, 4 to 19 pg g^−1^, 5 to 25 pg g^−1^, 100 to 1900 pg g^−1^, 710 to 6000 pg g^−1^, and ~ 0 to 12 pg g^−1^, respectively (Supplementary Table S2 and Fig. 3). In both records, no pronounced enrichment of PGEs was observed throughout the Turonian (Figs. 2, 3, 4). Chondrite-normalized PGE patterns show enrichment of P-PGEs (Pt and Pd), and Re, which have values close to those of the pelagic deep-sea sedimentary rocks and different from those of impact melt values (Fig. 4).

Age-corrected Os isotopic values (^187^Os/^188^Os_i_) were ~ 0.6 in the lowest Turonian and declined to 0.4 toward the Coniacian in both records (Figs. 2 and 3 and Supplementary Tables S1–S3). Because sedimentary rocks from both sites had low Re/Os, the effect of age correction was insignificant (less than 5%). Although three samples from the Bottaccione section (BTT 678, 704, and 728) had unradiogenic ^187^Os/^188^Os_i_ values (blue points in Fig. 2), their re-analyses did not reproduce the unradiogenic values. Therefore, we considered that the ^187^Os/^188^Os_i_ trends did not reflect hydrogenous information, but some other factors, such as local enrichment of unradiogenic material (i.e., extraterrestrial materials) in sedimentary rocks.

## Os isotopic variations during the Turonian

**Figure 5:**
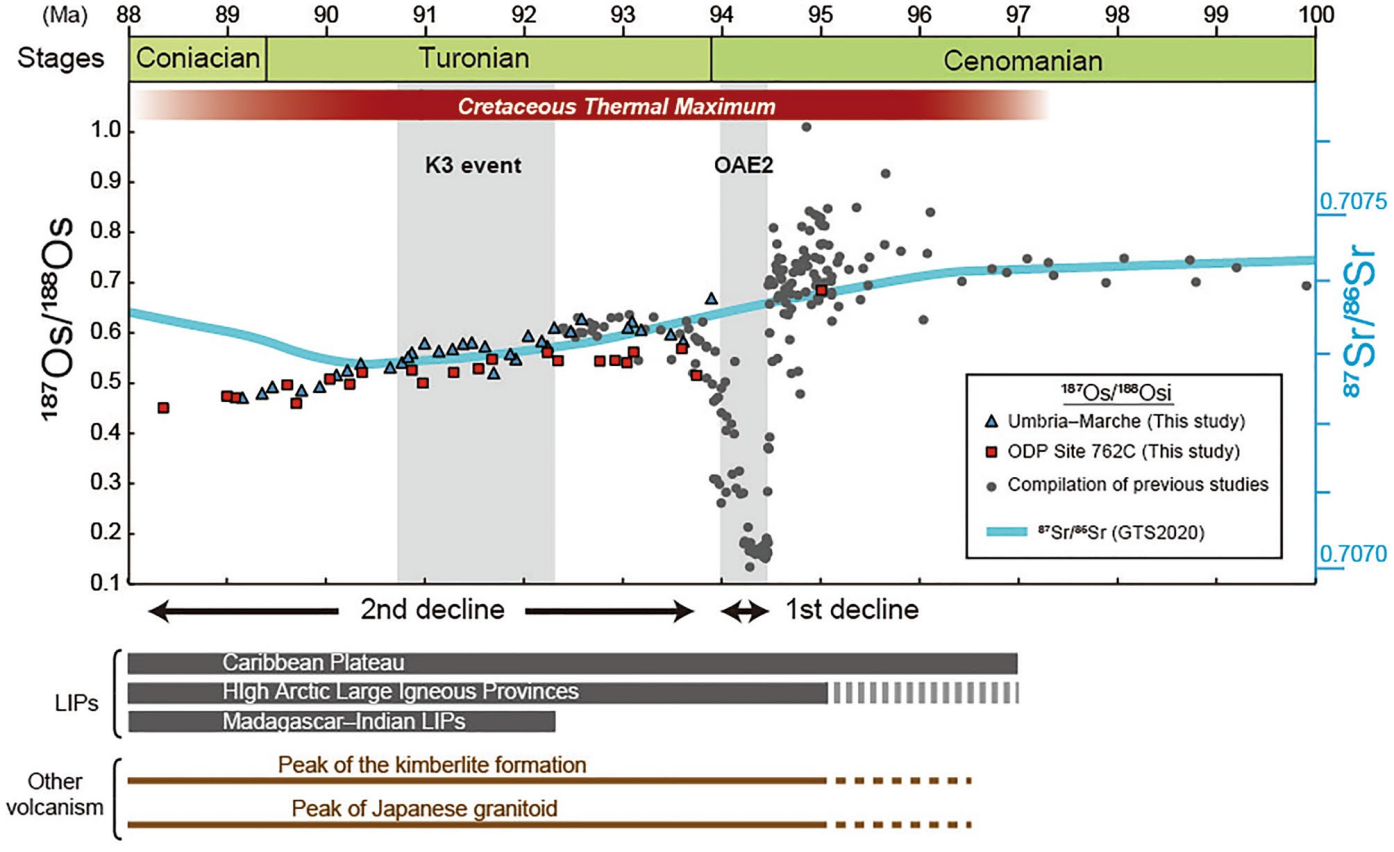
Compilation of Os isotopic data from the late Cenomanian to early Coniacian. Os isotopic data are from Refs.^31–34,72^, and this study. Sr isotopic data are from Ref.^73^. Ages of High Arctic Large Igneous Provinces are from Ref.^15^. Ages of the Caribbean Plateau are from Ref.^14^. Ages of the Madagascar Flood Basalt Province are from Refs.^13,18–20^. The ages of the kimberlite formation are based on Ref.^53^. The ages of Japanese granitoid are from Ref.^48^.

Previous studies have reported detailed Os isotopic variations from the late Cenomanian to the earliest Turonian^31–33^. During the late Cenomanian, the ^187^Os/^188^Os_i_ ratios were ~ 0.7 to 0.8^34^. During OAE2 (~ 93.9 Ma), ^187^Os/^188^Os_i_ ratios declined sharply toward mantle values (~ 0.2) (hereafter, 1st decline)^31–33^ (Fig. 5). Because the sedimentary age of OAE2 falls within the radiometric age range of Caribbean Plateau emplacement (97–70 Ma^14^), this unradiogenic shift has been interpreted to reflect the massive input of mantle-derived Os into the ocean through volcanic eruption associated with the formation of the Caribbean Plateau^31–33^. After OAE2, the ^187^Os/^188^Os_i_ ratios show a radiogenic shift toward the background value of ~ 0.6^31–33^ (Fig. 5).

Our newly obtained ^187^Os/^188^Os_i_ records from the Bottaccione section and ODP Site 762C reveal a continuous unradiogenic shift from ~ 0.6 to ~ 0.4 throughout the Turonian (hereafter, 2nd decline) (Fig. 5). Given that the ^187^Os/^188^Os_i_ variations, though derived from two totally different oceanic settings, are highly consistent (Fig. 5), we infer that our Os isotopic data reliably reflect paleo-seawater Os isotopic variations. Also, given the positive correlation between Os and other PGEs, our PGEs data should mainly reflect the hydrogenous fraction as well. However, similar to previously reported PGE data of pelagic sedimentary rocks, our data exhibit Ru- and Re-depleted patterns, in contrast to the patterns of modern seawater and the upper continental crust^30,39^ (Fig. 4). The Re and Ru depletion in the pelagic sedimentary sequence potentially reflects local modification of the seawater PGE patterns by preferential removal of Re under reducing oceanic conditions and of Ru under low-salinity conditions^35^. Based on the simple box model of Ref.^36^, the 2nd decline of Turonian marine Os isotope ratios can be explained by (1) a ~ 37-fold increase in the extraterrestrial Os flux, (2) a ~ 50% decrease in the input of radiogenic continental Os, or (3) a ~ twofold increase in the mantle-derived Os flux compared to the early Turonian background level.

Given th ^3^He-enrichment during the mid-Turonian K3 event, which has been interpreted due to the enhanced input of extraterrestrial material^21^, the first scenario seems plausible. However, we could not find a clear stratigraphic correlation between the ^187^Os/^188^Os variations and the estimated extraterrestrial ^3^He fluxes^21^ (Figs. 2 and 3). Also, our samples do not exhibit a significant enrichment of PGEs throughout the Turonian; this result suggests that the event of massive ^3^He input event did not significantly influence the seawater PGE cycles (Figs. 2, 3, and 5). Furthermore, Martin et al.^22^ reported that the concentrations of extraterrestrial spinels in the pelagic sediments did not change greatly throughout the Turonian interval. Considering these pieces of evidence, we infer that the input of extraterrestrial materials is a less plausible cause of the unradiogenic Os isotopic shift (2nd decline).

Another possibility is a decline in the continental weathering rate associated with the climate cooling. Indeed, previous studies reported monotonous ~ 3 °C decrease in the sea surface temperature in the Southern high latitude during the Turonian^6^. However, given the relationship between the temperature and weathering rate^37^, such decline in the temperature appear to be too small to account for the ~ 50% reduction in the continental weathering rate. An alternative explanation for the cause of the unradiogenic shift is the changes in the Os isotope ratio of the river water. Assuming the end-Cenomanian riverine ^187^Os/^188^Os ratio of 1.54^23^, a decline in the riverine ^187^Os/^188^Os ratios to 0.8 is required to explain the observed unradiogenic shift of seawater Os isotopic decline. However, considering ^187^Os/^188^Os values of most of the present major river water is above 1.0^23^, it is difficult to justify such large drop in riverine ^187^Os/^188^Os values.

The last scenario calls for an enhanced mantle-derived Os input throughout the Turonian age. Given that the oceanic crustal production rate at oceanic ridge during the Turonian was smaller than that during the previous stages^38^, enhanced unradiogenic Os inputs from oceanic ridges is not the cause of the 2nd decline. The radiometric ages of the Caribbean Plateau (97–70 Ma), the Madagascar Flood Basalt Province (92–66 Ma), and HALIP (97–80 Ma) roughly correspond to the timing of the 2nd Os isotopic decline^13–15,19,20,39,40^ (Fig. 5). Moreover, the 2nd decline of ^187^Os/^188^Os during the Turonian follows the large drop of ^187^Os/^188^Os (1st decline) during OAE2, which has been ascribed to volcanic events associated with the formation of the Caribbean Plateau^31,32^ (Fig. 5). Combining these pieces of evidence, we propose that the prolonged minor volcanism and hydrothermal activity associated with the emplacement of the Caribbean Plateau, the Madagascar Flood Basalt Province, or HALIP after the major volcanic pulse during OAE2 are the most plausible explanation for the 2nd decline of ^187^Os/^188^Os.

The onset of the unradiogenic shift of ^187^Os/^188^Os was almost simultaneous with the onset of the Sr isotopic ratio (^87^Sr/^86^Sr) decline (Fig. 5). The ^87^Sr/^86^Sr ratio of seawater, like that of Os, represents a balance between radiogenic continental Sr and unradiogenic mantle-derived Sr. Therefore, the unradiogenic shift of ^87^Sr/^86^Sr during the Turonian further supports an enhanced input of mantle-derived material into the ocean (Fig. 5). Intriguingly, around the lower Coniacian, the Sr and Os isotopic values start to show different trends; at that time, Sr isotopic values exhibit a radiogenic trend, while Os isotopic values continued to decline (Fig. 5).

One possible causes of this discrepancy between the Os and Sr isotopic variations would be an enhanced input of extraterrestrial materials. Because extraterrestrial materials can strongly influence PGE cycles on Earth, but they have less influence on the Sr cycle in the ocean, their input can explain the differences in the Os and Sr isotopic variations^41^. The ^3^He data and the low PGE concentrations during the Coniacian (Fig. 4) do not, however, support this interpretation^21^.

Another possible explanation is a change in riverine Os and Sr isotopic values: that is, lower ^187^Os/^188^Os ratios of riverine Os and/or higher ^87^Sr/^86^Sr ratios of riverine Sr in the Turonian–Coniacian. Indeed, previous studies have reported a large positive shift of δ^44^CA during the late Turonian to Coniacian that suggests a changes in continental weathering patterns associated with changes in paleogeography^42,43^. Therefore, the different trends of Os and Sr isotopic variations may indicate the change in the weathering pattern. For instance, previous studies have revealed that the drainage system of the Amazon changed during ~ Cenomanian to Maastrichtian, which was associated with the breakup of Gondowana^44^. Because rivers in the Amazon area discharge various lithology, including old Proterozoic cratons to the Paleozoic volcanic rocks, its change may have influenced the seawater Os and Sr cycles. At present, however, we do not have enough data to verify this possibility. Thus, further compilation of geological information and geochemical data is necessary to ascertain the cause of the discrepancy between the Os and Sr isotopic variations.

## Implications for the cause of the Turonian hot house world

One of the traditional hypotheses to explain the source of CO_2_ during the CTM is active oceanic crustal productions at spreading centers^45^. However, because the oceanic crustal production rate during the Turonian was lower than during previous ages^38^, this explanation seems unlikely.

Another possible source of CO_2_ was the active volcanic eruptions that formed oceanic plateaus. The ^187^Os/^188^Os ratios show a large unradiogenic shift during OAE2 at the end of the Cenomanian that is followed by a gradual ^187^Os/^188^Os decline throughout the Turonian (Fig. 5). These changes might reflect the massive volcanic events that formed the Caribbean Plateau and the Madagascar Flood Basalt Province as discussed in the previous section. These volcanic events might have released a large amount of CO_2_ that could have sustained the hot climate conditions of the CTM, the Earth’s warmest intervals of the Phanerozoic (Fig. 2). Some discrepancies exist, however, between the intensity of volcanic activity estimated from Os isotopic records and the temperature variations. The continuous decline of ^187^Os/^188^Os ratios of sedimentary rocks from the early Turonian to the Coniacian (2nd decline) might reflect the enhanced volcanic or hydrothermal activity associated with the emplacement of basaltic plateaus (i.e., the Caribbean Plateau, the Madagascar Flood Basalt Province, and HALIP) (Fig. 5). However, temperature during the mid-Cretaceous reached maximum values around the late Cenomanian to the early Turonian and then slightly decreased toward the Coniacian^6,8^ (Fig. 5). Given that the temperature slightly decreased as the volcanic and hydrothermal activity intensified, the input of CO_2_ through LIPs volcanism cannot completely explain the sustained hot-greenhouse world during the Turonian age.

Another possible scenario to explain the prolonged hot-greenhouse world is subaerial volcanic activity under the subaerial condition. It has been suggested that the length of the continental volcanic arc increased during the mid-Cretaceous and that this increased length enhanced the amount of volcanic CO_2_ emissions from subduction zones^46,47^. Moreover, the peak of Japanese granitoid formation occurred during the Cenomanian to Turonian (100–90 Ma), which roughly corresponds to the timing of the CTM^48^. Therefore, Matsumoto et al.^34^ have proposed that enhanced circum-Pacific volcanic activity was one of the triggers of the warm conditions during the Cretaceous. Although a large part of magma was solidified in the magma chamber, some of them erupted as rhyolite^49^. However, rhyolite typically exhibits very low Os concentration^50^, and thus, it may not have influenced the marine PGE cycles. Additionally, enhanced volcanic activity resulting in kimberlite formation may have been a major source of CO_2_ during the Turonian. Kimberlite is a highly carbonaceous volcanic rock (e.g., CO_2_ ~ 20 wt% solubility in the magmatic melt^51^), and kimberlite eruptions have a high ability to emit greenhouse gases^52^. Since the peak of the kimberlite formation occurred during the Cenomanian to Turonian (100–90 Ma)^53^, these volcanic events may have contributed to the CTM. Indeed, Patterson and Francis^52^ have suggested that kimberlite formation triggered early Cenozoic hyperthermal events. Although kimberlite exhibit high Os concentration (~ 0.03 to 8 ppb)^54^, it is composed of the cluster of small pipes (~ 10 ha)^53^ and its total volume is not significant enough to alter seawater PGE cycles. Therefore, we consider that the input of unradiogenic PGEs into the ocean through the weathering of kimberlite bodies was insignificant. Although further research on the volume of CO_2_ is essential, we suggest that a worldwide enhancement of volcanic activity, including of subaerial volcanism (kimberlite formation/circum-Pacific volcanic activity) and LIPs activity (Caribbean Plateau, Madagascar Flood Basalt Province, and HALIP), are the most probable candidates as the source of the CO_2_ supporting the CTM.

## Extraterrestrial events during Turonian

Farley et al.^21^ reported an increase in the extraterrestrial ^3^He flux during the Turonian. However, in this study, we did not find apparent declines of the ^187^Os/^188^Os and PGE enrichment that is observed in the massive meteorite impact event^55^ (Fig. 5). A possible explanation for this discrepancy between the present findings and those of Farley et al.^21^ is the IDP size. Basically, the influx of extraterrestrial PGEs onto the Earth is determined by the quantity of extraterrestrial material and the largest mass fraction is the IDPs with size of ~ 220 μm^56^. On the other hand, ^3^He in the sediments is originally derived from solar wind and exists on the surface of IDPs. Therefore, the total surface area of IDPs is a critical factor to determine the ^3^He flux on Earth^56^. Also, regarding extraterrestrial ^3^He, heating during the entry into the atmosphere is another important parameter for ^3^He flux on Earth^56,57^. During an influx of extraterrestrial materials into the atmosphere, friction with the atmosphere causes a surge in temperature. As the size of the extraterrestrial materials is larger, the friction with the atmosphere becomes more intense and the temperature becomes higher^58,59^. For example, in the case of IDPs larger than ~ 40 μm, their temperature during their entry into the atmosphere will exceed 800℃. This high temperature would cause most of helium contained in the particles to be released^59,60^. Indeed, the large-meteorite impact horizon at the Cretaceous–Paleogene boundary lacks ^3^He-enrichment, presumably because devolatilization during the impact event removed most ^3^He from the meteorites^50^. Based on the total surface area of IDP and the temperature during their entry into the atmosphere, the most important host phase of extraterrestrial ^3^He in sediment is considered to be fine-grained IDPs ranging from 3 to 35 μm in size^22,56^. However, the total mass fraction of such fine-grained IDPs is considerably smaller than that of larger fractions^57,61^. Thus, we consider that an increase in the flux of only fine-grained IDPs can explain both the ^3^He enrichment and the lack of Os and PGE signatures in the sediments during the K3 event.

Unlike the broad peaks of other ^3^He enrichment events during the Late Cretaceous to Cenozoic, the K3 event is composed of several spiky ^3^He concentration peaks^21^ (Fig. 2). In addition, the reproducibility of ^3^He concentrations in the sediments is very poor^21^. These enigmatic features were interpreted as the input of cosmic dust from the Moon^21^. Because of long exposure to solar wind and cosmic rays, lunar dust contains a large amount of ^3^He. Therefore, the input of even a very small amount of lunar dust can explain both the spikiness and poor reproducibility of ^3^He concentrations in the Turonian sediments^21^. Indeed, there are several large meteorite craters on the Moon that formed ~ 80–100 million years ago^62^, and these meteorite impacts may have related in large emissions of lunar dust into space. While ^187^Os/^188^Os of Moon rock is low (~ 0.12 to 0.2), their PGE concentrations were very low (0.5 ~ 65 ppt)^63^. Therefore, this meteorite impact event seems consistent with the lack of an Os isotopic declines and PGE enrichment in the sediments as well as with the non-chondritic PGE patterns (Figs. 2, 3, and 5). Here, typical cosmic ray exposure ages of most lunar meteorite that reached Earth are ~ 50 kyr^64^. Consequently, ~ 30 large asteroid impact events are expected to have occurred on the Moon to sustain the ^3^He-enrichment of the K3 event, which lasted ~ 1.5 Myr. Considering Earth has a stronger gravitational field than Moon, it must have experienced more impact events during the K3 event. However, our Os and PGE signals do not show any significant fluctuations during the K3 event, contradicting the possibility of the multiple asteroid impact events. Furthermore, chemical analysis of spinel grains (> 32 μm) contained in the Bottaccione section revealed no grains supporting a lunar origin^22^. Although Martin et al.^22^ did not exclude the possibility of an enhanced flux of only small size (< 32 μm) lunar IDPs, they alternatively proposed that resonance of the asteroid belt caused small asteroids to be ejected toward Earth, and some of these released ^3^He-rich regolith in Earth’s orbit. Since asteroid itself does not carry large amount of ^3^He^21^, this scenario cannot fully explain the long-term ^3^He-enrichment event. Showers of long-period comets, induced by gravitational perturbations of the Oort cloud, might also have delivered large amounts of small-sized cosmic particles to Earth^65^. This hypothesis is consistent with the lack of Os isotopic decline and PGE enrichment. Because the velocity of such long-period comets with perihelia less than 1.2 AU tends to be very high^65–67^, particles derived from these comets usually reach a very high temperature during their entry into the Earth’s atmosphere^68^, potentially releasing the He contained therein. However, the velocities of very fine cometary dusts decrease before entering the atmosphere because of the Poynting-Robertson drag^67^ and most of them do not experience melting during the entry into the atmosphere^58^. Therefore, it is possible for fine cometary dust to retain the ^3^He during the entry onto the Earth. We concluded that the fine-cosmic (< 32 μm) dust released from the showers of long-period comets could be responsible for the lack of Os and PGE signatures and enrichment of ^3^He during the K3 event. Nevertheless, further research on small-sized extraterrestrial materials (< 32 μm) and the host phases of ^3^He in Turonian sediments is essential to test this and other hypotheses.

## Conclusions

Here, we presented Os isotopic and PGE variations in Turonian sedimentary rocks and identified a monotonic decline of Os isotopic values (2nd decline) that may represent the enhanced input of mantle-derived Os released during the volcanic events associated with the formation of the Caribbean Plateau and/or the Madagascar LIPs. The timing of the warmest climate interval, the CTM, does not however correspond to the unradiogenic Turonian peak in the Os isotopic record (2nd decline). Therefore, the extremely hot Cretaceous world cannot be solely explained by active volcanic degassing associated with LIP formation; rather, additional processes, such as subaerial volcanic activity in subduction zones or kimberlite formation, must provide part of the explanation. Because Os isotope ratios do not show an unradiogenic shift, and PGE concentrations do not exhibit a pronounced enrichment, an influx of small IDPs to the Earth’s surface, possibly from the long-period comet shower can be inferred at the time of the ^3^He enrichment during the mid-Turonian K3 event. Our findings highlight the different behaviors of ^3^He and PGE information in the sedimentary rocks during the input of fined-grained extraterrestrial materials.

## Methods

Sedimentary rocks samples were trimmed and ultrasonically cleaned. After drying, powdered samples (~ 0.5 g) were spiked with a solution enriched in ^185^Re, ^190^Os, ^191^Ir, ^99^Ru, ^196^Pt, and ^105^Pd and sealed in a quartz glass tube with 4 mL of inverse aqua regia. The tubes were heated at 230℃ for 48 h. After centrifugation, Os in the supernatants was separated and purified by CCl_4_ extraction, HBr back extraction, and microdistillation. The Os concentration and isotopic composition were determined by negative thermal ionization mass spectrometry (N-TIMS, TRITON, Thermo Fisher) at the Japan Agency for Marine-Earth Science and Technology (Japan). Re and other PGEs were separated and purified through an anion exchange resin (Muromac AG1-X8, 100–200 mesh) and a cation exchange resin (BioRad AG 50W-X8, 200–400 mesh). PGE concentrations were measured by inductively coupled plasma mass spectrometry (ICP-MS, Thermo Element XR, Thermo Fisher at the University of Tokyo (Japan). All data were collected with respect to the values of procedural blanks (0.12 ± 0.05 pg for Os with ^187^Os/^188^Os = 0.2 ± 0.04; 1.4 ± 0.1/0.6 ± 0.2 pg for Re; 1.4 ± 0.9 pg for Ir; 0.5 ± 0.5 pg for Ru; 37.4 ± 21.6 and 7.1 ± 3.6 pg for Pt; and 1.4 ± 1.3 pg for Pd). The detailed methods are described in Refs.^29,30^.

### Supplementary Information

Supplementary Information.

## Data Availability

All data generated in this study are included in the Supplementary file.
